# Alexithymia predicts maladaptive but not adaptive emotion regulation strategies in adolescent girls with anorexia nervosa or depression

**DOI:** 10.1186/s40337-019-0271-1

**Published:** 2019-11-29

**Authors:** Anca Sfärlea, Sandra Dehning, Lena Katharina Keller, Gerd Schulte-Körne

**Affiliations:** 1 Department of Child and Adolescent Psychiatry, Psychosomatics and Psychotherapy, University Hospital, LMU Munich, Pettenkoferstr. 8a, 80336 Munich, Germany; 2 Faculty of Medicine, Institute of Medical Psychology, LMU, Munich, Germany

**Keywords:** Adolescence, Emotion regulation, Alexithymia, Anorexia nervosa, Major depression

## Abstract

**Background:**

Among adolescent girls, anorexia nervosa (AN) and major depression (MD) are common and often comorbid mental health problems. Both disorders are characterised by difficulties in recognising and verbalising (alexithymia) as well as regulating one’s emotions, but research in adolescent patients is scarce and little is known about the relation between alexithymia and difficulties in emotion regulation. The aims of this study were to investigate alexithymia and emotion regulation skills in adolescents with AN, adolescents with MD, and healthy adolescents, and to determine whether alexithymia functions as a predictor for emotion regulation skills.

**Methods:**

Emotion regulation strategies, alexithymia, and depressive symptoms were assessed by questionnaire measures in 12–18 year old girls with AN (*n* = 26), girls with MD (*n* = 25), and healthy girls (*n* = 35). Groups were compared with respect to the these variables and multiple regression analyses were calculated separately for adaptive and maladaptive emotion regulation strategies in order to examine if alexithymia predicted emotion regulation over and above age and depressive symptoms.

**Results:**

Girls with AN or MD both reported using adaptive emotion regulation strategies less frequently and maladaptive emotion regulation skills more frequently as well as higher levels of alexithymia compared to healthy girls. Alexithymia positively predicted maladaptive emotion regulation strategies, while depressive symptoms negatively predicted adaptive emotion regulation strategies.

**Conclusions:**

The results suggest that different mechanisms may underlie the lack of adaptive and the surplus of maladaptive emotion regulation strategies in adolescent psychiatric patients.

## Plain English summary

Anorexia nervosa and major depression are common mental health problems among adolescent girls. Many people who suffer from these disorders also have difficulties dealing with their emotions: they struggle recognising and talking about their emotions (a psychological characteristic called “alexithymia”) as well as regulating their emotions appropriately. However, as most of this research on alexithymia and emotion regulation has been conducted in adult samples, it is not known if the results can also be applied to adolescent patients. Furthermore, little is known about the relationship between alexithymia and emotion regulation. Therefore, our study assessed alexithymia and emotion regulation skills in three groups of adolescent girls: girls with anorexia nervosa, girls with major depression, and healthy girls. Both patient groups reported higher levels of alexithymia and made less use of adaptive emotion regulation strategies and more use of maladaptive emotion regulation strategies, compared to healthy girls. Alexithymia was associated with both adaptive emotion regulation strategies (negatively) and maladaptive emotion regulation strategies (positively), but explained additional variance only in maladaptive emotion regulation strategies, while the use of adaptive emotion regulation strategies was explained by depressive symptoms. Our results add to the small body of research on alexithymia and emotion regulation in adolescent psychiatric patients and suggest that different mechanisms may underlie the lack of adaptive and the surplus of maladaptive emotion regulation strategies in these patients.

## Background

Among adolescent girls, anorexia nervosa (AN) and major depression (MD) are common mental health problems (e.g., [1, 2]) that frequently occur comorbidly (e.g., [3, 4]). For adolescents with AN and MD, regulating emotions in a healthy way seems to be difficult (e.g., [5, 6]), but research in adolescent patients is limited [7, 8]. Emotion regulation difficulties can persist into adult age (e.g., [9, 10]), can contribute to the maintenance of the disorders [11, 12] and seem to be not specific for certain mental disorders but rather a transdiagnostic characteristic (e.g., [13, 14]).

Strategies to regulate emotions can be classified into adaptive (e.g., acceptance, problem-solving, reappraisal) and maladaptive strategies (e.g., rumination, avoidance, suppression) [8, 15]. They usually develop from infancy to adulthood (e.g., [16, 17]), with a so-called “maladaptive shift” occurring between 12 and 15 years [17, 18]. At this age, the use of adaptive emotion regulation strategies decreases while the use of maladaptive strategies increases. This can be explained, i.a., by neuroendocrinological maturation [19–21] and the transition from parent-guided to self-guided emotion regulation [22]. This “maladaptive shift” is not specific for adolescents suffering from mental disorders but typically occurs in adolescents; however, it may explain why adolescence is a particularly vulnerable period for the development of mental disorders [23, 24].

A psychological construct related to emotion dysregulation is alexithymia, which literally means “no words for mood” and refers to difficulties in identifying and describing one’s emotions (e.g., [25, 26]). While numerous studies have linked alexithymia to various mental disorders including AN and MD in adults (see e.g., [26–28]), less is known about alexithymia in adolescent patients [29]: Alexithymia has been found to be increased in adolescents with AN (e.g., [30, 31]) and to be related to depressive symptoms in adolescents [32] but has not been investigated in adolescents with MD [33]. However, alexithymia has been found to be associated with non-suicidal self-injury (e.g., [34, 35]) and to characterise adolescents with borderline personality disorder [36], indicating that it plays an essential role in disordered emotional functioning in adolescence.

Alexithymia has often been considered as a part of deficient emotion regulation (e.g., [25]) but it could be speculated that the ability to recognise one’s own emotional reactions is actually a necessary *premise* for adaptive emotion regulation (e.g., [29, 37, 38]): individuals who struggle with detecting and naming their emotional state in a specific situation may find it particularly difficult to choose an appropriate strategy to regulate this emotional state as this requires understanding of the specific emotional elicitors as well as the elicited emotions [39]. This idea is in line with results from a study in adult inpatients with AN that found alexithymia at admission to predict emotion dysregulation at discharge [40], suggesting that alexithymia might interfere with therapeutic attempts to improve emotion regulation. Relationships between alexithymia and emotion regulation have also been reported in a mixed group of adolescent inpatients [29] as well as student samples [39, 41, 42] but the exact mechanisms relating alexithymia to emotion regulation are not fully understood. Research in adolescent psychiatric patients is particularly scarce and, so far, no study has examined to what extent alexithymia predicts emotion regulation in adolescents separately for adaptive and maladaptive emotion regulation strategies. This seems important considering that different emotion regulation strategies have been found to be differently related to alexithymia in young adults [41].

Therefore, the present study was designed to investigate the role of alexithymia for emotion regulation in two groups of adolescent psychiatric patients: girls with AN and girls with MD, compared to healthy girls. First, we aimed to elucidate if alexithymia and emotion regulation skills differ between adolescent girls with AN or MD and healthy girls. Second, we wanted to determine whether alexithymia functions as a predictor for emotion regulation skills, over and above age and depressive symptoms, which are known to be related to emotion regulation (e.g., [8, 43]). This would be in line with the notion that the ability to recognise one’s own emotional reactions represents a premise for adaptive emotion regulation. In line with the literature (e.g., [5, 6, 31, 32]), we expected adolescents with AN and MD to report higher alexithymia scores as well as to use less adaptive and more maladaptive emotion regulation strategies. Furthermore, as alexithymia has been found to be related to emotion regulation difficulties (e.g., [29, 42]), we expected alexithymia to be a negative predictor for adaptive emotion regulation strategies and a positive predictor for maladaptive emotions regulation strategies.

## Methods

### Participants

The present data were collected within a study on emotional face processing in AN [44, 45]. The study sample consisted of 35 healthy girls, 26 girls with AN, and 25 girls with MD, aged 12 to 18 years.^1^ Girls with AN and MD were inpatients or outpatients from the Department of Child and Adolescent Psychiatry, Psychosomatics and Psychotherapy of the University Hospital Munich, Germany. Girls in the healthy control (HC) group were recruited through previous studies in which they had participated as HCs. Written information about the study together with an invitation to take part was sent to their families. Other HC girls were recruited through local advertisements. All interested HC participants were initially screened by asking their parents about any mental health problems of the adolescent before inviting them to our lab.

Psychiatric diagnoses were assessed in all participants using a standardised, semi-structured clinical interview (Kinder-DIPS [47]). The Kinder-DIPS is a well-established German diagnostic interview that allows diagnosis of a wide range of axis I disorders in children and adolescents and shows good inter-rater reliabilities for all diagnostic clusters [48]. The interviews were conducted and evaluated by trained interviewers with both, participants and their parents. Exclusion criteria for all participants were IQ < 85 (measured via the CFT-20-R [49]), current neurological disorders, pervasive developmental disorders, attention deficit and hyperactivity disorder, schizophrenic disorder, and bipolar disorder.

Girls were included in the HC group if they did not meet criteria for any current or past axis I disorder as assessed via the Kinder-DIPS.

Girls were included in the AN group if they met criteria for AN according to ICD-10 (F50.0 [50]) and had a body mass index (BMI) on or below the 3rd age-corrected percentile according to KiGGS [51]. Sixteen of the included AN patients fulfilled criteria for at least one comorbid condition, including MD, conduct disorder, and anxiety disorders. Depression was the most frequent comorbid diagnosis (*n* = 13) and had developed secondary to the eating disorder in most cases.^2^

Girls were included in the MD group if they currently met criteria for an episode of MD (F32 or F33 in the ICD-10 [50]) and did not report any symptoms of an eating disorder. Within the MD group, 10 patients met criteria for one or more comorbid anxiety disorders.

### Measures

Depressive symptoms were assessed with the German version of the Beck Depression Inventory-II (BDI-II [52]), a 21-item self-report questionnaire that allows valid assessment of depressive symptoms in adolescents [53]. Internal consistency in our sample was excellent (Cronbach’s α = .95).

To measure alexithymia, the German version of the Toronto Alexithymia Scale (TAS [54]) was used. The TAS is a self-report questionnaire for the valid assessment of three dimensions of alexithymia: (1) difficulty identifying feelings, (2) difficulty describing feelings, and (3) externally oriented thinking. Eighteen items were rated on a five-point scale and summed up to a total alexithymia score. Internal consistency in our sample was good (Cronbach’s α = .83).

The German questionnaire on emotion regulation in children and adolescents (FEEL-KJ [55]) was administered to measure emotion regulation skills. The 90-item self-report questionnaire assesses the habitual use of adaptive (acceptance, cognitive problem solving, distraction, forgetting, humour enhancement, problem-oriented action, reevaluation) and maladaptive strategies (aggressive behavior, giving up, rumination, self-devaluation, withdrawal) to regulate anxiety, fear, and sadness in children and adolescents and has proven to be valid and reliable [56]. Each item (e.g., “I try to change what makes me angry”, “I cannot get it out of my head”) is rated on a five-point scale according to how often this strategy is applied to regulate each of the emotions. Sum scores for adaptive and maladaptive strategies across all emotions were calculated. Internal consistencies in our sample were good (Cronbach’s αs ≥ .89).

### Data analysis

Statistical analyses were conducted with SPSS 24. To assess group differences one-way analyses of variance (ANOVAs) and follow-up *t*-tests were conducted. Hierarchical linear regressions were used to determine the extent to which variations in alexithymia explain variations in emotion regulation in addition to known predictors: In a first step, age and depressive symptoms were included as predictors; in a second step alexithymia was added. Outcome variables were adaptive as well as maladaptive emotion regulation strategies. The significance level (*p* = .05, two-tailed) was adjusted according to the Bonferroni-Holm procedure [57] when performing multiple post-hoc comparisons.

## Results

### Group comparisons

Characteristics of the three groups are presented in Table 1. The groups were comparable in terms of age and IQ but differed, as expected, regarding BMI (with the AN group having a lower BMI and age-corrected BMI-percentile than the MD and HC groups; *t*s ≥ 6.0, *p*s < .001, *d*s ≥ 1.7) and depressive symptoms (with both patient groups reporting more depressive symptoms than the HC group: *t*s ≥ 8.4, *p*s < .001, *d*s ≥ 2.4; while not differing from each other: *t*_49_ = 1.1, *p* > .1). Both patient groups reported more pronounced alexithymia than the HC group (*t*s ≥ 7.3, *p*s < .001, *d*s ≥ 2.0), but did not differ from each other (*t* < 1). AN and MD groups reported to use adaptive emotion regulation strategies less frequently (*t*s ≥ 2.8, *p*s ≤ .008, *d*s ≥ 0.7) and maladaptive emotion regulation strategies more frequently (*t*s ≥ 5.8, *p*s < .001, *d*s ≥ 1.0) than the HC group, while not differing from each other (*t*s ≤ 1.6, *p*s > .05). Emotion regulation strategies and alexithymia were highly correlated with each other as well as with depressive symptoms, but not related to age (Table 2).

**Table 1:** Demographic and clinical characteristics as well as emotional skills of the study sample

	HC	AN	MD	ANOVA			post-hoc tests
	*n* = 35	*n* = 26	*n* = 25				
	*M* (*SD*)	*M* (*SD*)	*M* (*SD*)	*F*_2,83_	*p*	*η*_*p*_^*2*^	
Age	15.2 (1.8)	15.2 (1.7)	14.7 (1.6)	1.0	n.s.	.03	
IQ	105.9 (10.3)	106.6 (15.0)	110.9 (12.5)	1.2	n.s.	.03	
BMI	19.8 (2.0)	15.4 (1.2)	21.4 (4.9)	28.0	< .001	.40	AN < HC = MD
BMI-percentile (age-corrected)	36.0 (22.0)	0.8 (1.2)	49.2 (32.4)	32.5	< .001	.44	AN < HC = MD
Depressive symptoms	4.4 (3.8)	23.8 (9.7)	27.3 (13.2)	55.9	< .001	.57	HC < AN = MD
Alexithymia	40.4 (6.2)	54.7 (8.1)	57.1 (8.3)	45.1	< .001	.52	HC < AN = MD
Emotion regulation strategies							
Adaptive strategies	131.6 (25.9)	111.8 (30.0)	104.4 (25.6)	8.2	.001	.16	HC > AN = MD
Maladaptive strategies	76.5 (15.7)	92.4 (15.0)	99.2 (13.8)	18.4	< .001	.31	HC < AN = MD

*HC* Healthy controls, *AN* Anorexia nervosa, *MD* Major depression, *IQ* Intelligence quotient, *BMI* Body mass index, *M* Mean, *SD* Standard deviation; post-hoc *t*-tests were all significant with *p* < .01

**Table 2:** Pearson-Correlations

	Age	Depressive symptoms	Alexithymia	Adaptive emotion regulation strategies
Depressive symptoms	−.14			
Alexithymia	.00	.80*		
Adaptive emotion regulation strategies	.14	−.43*	−.37*	
Maladaptive emotion regulation strategies	.03	.58*	.66*	−.42*

*p < .001

### Prediction of emotion regulation strategies

The hierarchical regression revealed that age and depressive symptoms accounted for a significant proportion of variance of adaptive emotion regulation strategies (*F*_2,83_ = 9.9, *p* < .001, *R*^*2*^ = .19). Adding alexithymia to the model did not increase the proportion of explained variance significantly (*p* > .1). Depressive symptoms were a significant negative predictor for adaptive emotion regulation strategies, while age and alexithymia were not (Table 3).

**Table 3:** Results of the regression analyses predicting emotion regulation strategies as a function of age, depression, and alexithymia

	*B*	*SE for B*	*95% CI for B*	*β*	*t*	*p*
Adaptive strategies						
STEP 1:						
Age	1.4	1.7	[−2.0, 4.8]	.08	< 1	n.s.
Depressive symptoms	−0.9	0.2	[−1.3, − 0.5]	−.42	−4.2	< .001
STEP 2:						
Age	1.6	1.8	[−1.9, 5.1]	.09	< 1	n.s.
Depressive symptoms	−0.7	0.4	[−1.5, − 0.0]	−.35	−2.1	.043
Alexithymia	−0.2	0.5	[−1.2, 0.7]	−.08	< 1	n.s.
Maladaptive strategies						
STEP 1:						
Age	1.1	0.9	[−0.7, 3.0]	.11	1.2	.230
Depressive symptoms	0.8	0.1	[0.5, 1.0]	.59	6.6	< .001
STEP 2:						
Age	0.5	0.9	[−1.3, 2.3]	.05	< 1	n.s.
Depressive symptoms	0.2	0.2	[−0.2, 0.6]	.16	1.2	.253
Alexithymia	0.9	0.2	[0.4, 1.3]	.53	3.8	< .001

For maladaptive emotion regulation strategies, the analysis revealed that age and depressive symptoms accounted for a significant proportion of variance (*F*_2,83_ = 21.9, *p* < .001, *R*^*2*^ = .35). Adding alexithymia to the model increased the explained variance significantly (Δ*R*^*2*^ = .10, *p* < .001) resulting on a total of 44% of variance explained (*F*_3,82_ = 21.6, *p* < .001, *R*^*2*^ = .44). Alexithymia was a significant positive predictor for maladaptive emotion regulation strategies in the final model, while age and depressive symptoms were not (Table 3).

In both regression analyses tolerance values were > .2, indicating that multicollinearity was not a problem [46].

## Discussion

The present study investigated alexithymia and emotion regulation in adolescent girls with AN or MD and healthy girls. Both patient groups reported higher alexithymia scores as well as less adaptive and more maladaptive emotion regulation strategies than HCs. Alexithymia was a strong positive predictor for maladaptive emotion regulation strategies while adaptive emotion regulation strategies were negatively predicted by depressive symptoms.

In line with our expectations, we found that girls with AN as well as girls with MD displayed higher alexithymia scores and reported using adaptive emotion regulation strategies less frequently and maladaptive emotion regulation strategies more frequently compared to HCs. This is consistent with prior studies in adolescents (e.g., [5, 6, 30–32, 58–60]) and adds to the small body of literature on alexithymia and emotion regulation in adolescent psychiatric patients. The two patient groups did not differ from each other, suggesting that both, difficulties in recognising and verbalising as well as regulating one’s emotions do not characterise AN or MD specifically but might represent transdiagnostic factors already in adolescence, which is in line with prior studies on alexithymia [29] and emotion regulation [61] in children and adolescents with different mental disorders. However, different diagnostic groups might differ with respect to the specific emotion regulation strategies,^3^ so future studies should investigate different adaptive and maladaptive emotion regulation strategies separately in larger samples of adolescents with AN and MD, as this will allow a more differentiated understanding of emotion regulation difficulties in these groups.

The regression analyses showed that alexithymia was a significant positive predictor for maladaptive regulation strategies, but adaptive emotion regulation strategies were negatively predicted only by depressive symptoms. This is in line with studies in healthy adults that found alexithymia to be related to the maladaptive emotion regulation strategy suppression, but not the adaptive strategy reappraisal ([41, 62, 63]; but see also [42]). Alexithymia being a strong predictor for maladaptive emotion regulation strategies suggests that uncertainty about one’s emotions enhances the use of inadequate strategies to regulate them. Venta et al. [29] suggested that this relation might be explained by experiential avoidance: On the one hand, avoidance of aversive experiences may interfere with learning how to use emotional language in a pragmatic way, thereby contributing to alexithymia, on the other hand, it may prevent individuals to learn how to deal with difficult situations, leading to reduced emotion regulation abilities. Alternatively, one could suspect that the difficulties in perceiving and verbalising one’s feelings might impede individuals’ ability to reflect upon their feelings and to generate emotion-specific, appropriate reactions, thereby fostering maladaptive behaviour like aggression or withdrawal [39].

The result that depressive symptoms were a negative predictor for adaptive emotion regulation strategies on the other hand, might be accounted for by different explanations. One is the decrease of positive activity, which is one of the core symptoms of depressive disorders [50] and might involve reduced availability of adaptive strategies as distraction or humour enhancement. Another possible explanation are cognitive biases for negative information that are associated with depression [64, 65] and presumably render revaluation of emotion-eliciting situations difficult. However, both explanations are highly speculative and the relationship between emotion regulation and depressive symptoms is likely of bidirectional nature, as difficulties in emotion regulation are both, a risk factor for depression (e.g., [66]) as well as a result of depressive symptomatology (the latter seems to apply particularly to the adaptive strategy reappraisal [67]).

Of note, in the first step of the regression, depressive symptoms predicted the use of both adaptive as well as maladaptive emotion regulation strategies, as expected (e.g., [8]). However, entering alexithymia into the regression model reduced the influence of depressive symptoms. This presumably results from the high correlation between depressive symptoms and alexithymia in our sample (which is in line with e.g., [58, 68]). It is arguable if alexithymia can be assessed validly in depressed samples (e.g., [69]), as it is still unknown whether alexithymia is a state that depends on depressive symptoms and decreases as these remit (e.g., [70]) or rather a stable personality trait (e.g., [71]). Our results show that in spite of sharing a great amount of variance, depressive symptoms and alexithymia have differential influence on adaptive and maladaptive emotion regulation strategies in adolescents, suggesting that different mechanisms may underlie the lack of adaptive and the surplus of maladaptive emotion regulation strategies in adolescent psychiatric patients. As the present study was the first to investigate the interplay between alexithymia and emotion regulation in adolescent girls with AN and MD, further studies that also include weight-restored or remitted patients are necessary to further disentangle the differential influence of depressive symptoms and alexithymia on emotion regulation. For example, if the use of adaptive strategies depends mainly on depressive symptoms and the use of maladaptive strategies depends more on alexithymia, one could suspect that the lack of adaptive strategies improves with remission of depressive symptoms while the use of maladaptive strategies might remain – at least to some extent – increased, unless a decrease in alexithymia occurs.

An important limitation of our study results from our AN and MD samples reporting a comparable amount of depressive symptoms.^4^ Considering the close connection between depressive symptoms and alexithymia, this might have masked differences in alexithymia between these groups (depressive symptoms have been found to explain alexithymia in AN partially but not entirely [68]). It might also have prevented us from finding differences in emotion regulation like Brockmeyer et al. [9] reported between adults with AN (without comorbid depression) and MD (with both patient groups showing greater emotion regulation difficulties than HCs but MD patients reporting even greater difficulties than AN patients). In addition, a considerable proportion of patients in both groups had comorbid anxiety disorders. Therefore, we cannot draw conclusions that are specific for girls with AN or MD from the group comparisons.

Another limitation is the size and composition of the study sample. As mentioned above, the limited sample size prevented us from investigating group differences in different emotion regulation strategies. The heterogenity of the patient groups, which comprised in- a well as outpatients with different disorder severities and comorbid conditions, might have further contributed to the inability to detect differences between AN and MD patients.

The interpretation of our results is also limited by the fact that our study relies solely on self-report measures. Adolescents’ self-reported emotion regulation strategies might not reflect the actual behaviour when encountering negative emotions [73] and reporting to use adaptive emotion regulation strategies less frequently may not necessarily implicate less successful emotion regulation (e.g., [74]). Future studies should investigate both, self-reported emotion regulation skills and performance in emotion regulation tasks to elucidate if adolescent AN and MD patients indeed are less successful in regulating their emotions or if they underestimate their actual abilities.

Importantly, due to the cross-sectional design of the study, we cannot draw conclusions regarding causality and temporal relationships: it is possible that the inability to identify one’s emotions (i.e., being alexithymic) indeed precedes and is a cause of maladaptive emotion regulation but it is also possible that another factor influences both, difficulties in emotion regulation and alexithymia. Longitudinal studies could shed light on these possibilities as well as the aforementioned question of stability of the relationship between alexithymia and emotion regulation in remission.

Our study has important clinical implications. Our result of alexithymia explaining a substantial part of variance in maladaptive emotion regulation, together with the finding that alexithymia has a negative impact on treatment outcome (e.g., [75, 76]), presumably, i.a., by interfering with therapeutic attempts to improve emotion regulation [40], stresses the importance of addressing adolescent patients’ abilities to identify and express their emotions in order to improve their emotion regulation abilities. Given that improving emotion regulation has been found to improve treatment outcomes in patients with AN and MD (e.g., [11, 12, 77]) and considering that development of functional emotion regulation skills is an essential task in adolescence (e.g., [23]), young patients may especially benefit from therapies focused on their emotional competencies.

## Conclusion

Our study was the first to investigate the interplay between alexithymia, depressive symptoms, and adaptive as well as maladaptive emotion regulation strategies in adolescent girls with AN and MD in comparison to healthy girls. The results replicate findings of more pronounced alexithymia as well as less adaptive and more maladaptive emotion regulation strategy use in the patient groups. Furthermore, they suggest that alexithymia and depressive symptoms have differential influence on adaptive and maladaptive emotion regulation strategies, partially supporting the idea that the ability to recognise and describe one’s own emotions might be a necessary premise for successful emotion regulation. However, additional studies are needed to elucidate this topic and other explanations should also be considered.

## Data Availability

The datasets generated and analysed during the current study are not publicly available because no consent for making the data publicly available was collected from the participants. However, the data are available from the corresponding author on reasonable request.

## Abbreviations

AN: Anorexia nervosa
ANOVA: Analysis of variance
BDI-II: Beck Depression Inventory-II
BMI: Body mass index
FEEL-KJ: Fragebogen zur Erhebung der Emotionsregulation bei Kindern und Jugendlichen [questionnaire on emotion regulation in children and adolescents]
HC: Healthy control
IQ: Intelligence quotient
Kinder-DIPS: Diagnostisches Interview bei psychischen Störungen im Kindes- und Jugendalter [diagnostic interview for mental disorders in childhood and adolescence]
MD: Major depression
TAS: Toronto Alexithymia Scale

## Footnotes

1 Only girls who completed all questionnaires were included. One girl was identified as outlier in the regression analysis (according to [46]) and thus excluded from all analyses.

2 Participants with comorbid MD were not excluded as this would have led to a non-representative sample given the high comorbidity between AN and MD (e.g., [3]) and AN patients not meeting criteria for MD also showing substantially elevated depressive symptoms.

3 Explorative analyses of our data indicated that there might be differences for specific emotion regulation strategies between AN and MD patients, particularly for the maladaptive strategy *giving up* which seemed to be employed more often by MD patients than by AN patients. However, our restricted sample size does not allow confident interpretation of these analyses, so the investigation of different emotion regulation strategies separately remains subject to future research.

4 Of note, some of the depressive symptoms in AN patients may be accounted for by malnutrition (e.g., [72]).
